# Supplementary material for: Hot spot prediction in protein-protein interactions by an ensemble system
Source: BMC Syst Biol. 2018 Dec 31;12(Suppl 9):132. doi: 10.1186/s12918-018-0665-8 (PMC6311905; doi:10.1186/s12918-018-0665-8)
Supplement: Supplementary file 2 — All relevant MATLAB code. A simple Matlab implement of our predictor. (ZIP 19,742 kb) [file 12918_2018_665_MOESM2_ESM.zip › Additional file 2/Read ASA/ASA Property/SKEMPI/2QJBC.txt]

 NetSurfP - Protein Surface Accessibility and Secondary Structure Predictions

  

|  |  |  |
| --- | --- | --- |
|  |  | NetSurfP - Protein Surface Accessibility and Secondary Structure Predictions  Technical University of Denmark |

  


---

```
# For publication of results, please cite:
# A generic method for assignment of reliability scores applied to solvent accessibility predictions.
# Bent Petersen, Thomas Nordahl Petersen, Pernille Andersen, Morten Nielsen and Claus Lundegaard
# BMC Structural Biology 2009, 9:51 doi:10.1186/1472-6807-9-51
#
# Column 1: Class assignment - B for buried or E for Exposed - Threshold: 25% exposure, but not based on RSA
# Column 2: Amino acid
# Column 3: Sequence name
# Column 4: Amino acid number
# Column 5: Relative Surface Accessibility - RSA
# Column 6: Absolute Surface Accessibility
# Column 7: Z-fit score for RSA prediction
# Column 8: Probability for Alpha-Helix
# Column 9: Probability for Beta-strand
# Column 10: Probability for Coil
E T  2QJBC                  1    0.859 119.102   0.360   0.003   0.003   0.994
B L  2QJBC                  2    0.261  47.734  -0.775   0.005   0.045   0.951
E P  2QJBC                  3    0.367  52.006  -1.150   0.018   0.088   0.893
E F  2QJBC                  4    0.281  56.457  -0.379   0.021   0.279   0.699
B L  2QJBC                  5    0.067  12.341  -1.188   0.003   0.718   0.279
E K  2QJBC                  6    0.335  69.012   1.182   0.001   0.900   0.099
B C  2QJBC                  7    0.024   3.370   0.170   0.001   0.900   0.099
B Y  2QJBC                  8    0.253  54.087   1.185   0.001   0.900   0.099
B C  2QJBC                  9    0.019   2.710   0.833   0.004   0.616   0.381
E S  2QJBC                 10    0.307  35.934   0.535   0.004   0.197   0.799
E H  2QJBC                 11    0.696 126.511  -1.910   0.019   0.141   0.840
E H  2QJBC                 12    0.404  73.433   0.247   0.018   0.088   0.893
B C  2QJBC                 13    0.037   5.223  -1.240   0.018   0.047   0.935
E P  2QJBC                 14    0.595  84.459  -0.403   0.018   0.047   0.935
E E  2QJBC                 15    0.702 122.570  -1.759   0.018   0.019   0.964
E D  2QJBC                 16    0.545  78.477  -1.641   0.018   0.019   0.964
E A  2QJBC                 17    0.456  50.295  -1.337   0.018   0.019   0.964
E I  2QJBC                 18    0.510  94.406  -0.611   0.018   0.047   0.935
E N  2QJBC                 19    0.434  63.567  -1.117   0.018   0.047   0.935
E N  2QJBC                 20    0.298  43.671  -0.764   0.018   0.088   0.893
E T  2QJBC                 21    0.324  44.869   0.868   0.022   0.359   0.619
B C  2QJBC                 22    0.048   6.725  -0.304   0.022   0.552   0.426
E I  2QJBC                 23    0.415  76.794   1.016   0.069   0.386   0.545
E T  2QJBC                 24    0.496  68.809  -0.673   0.064   0.216   0.721
E N  2QJBC                 25    0.601  87.928  -0.581   0.113   0.043   0.844
B G  2QJBC                 26    0.273  21.501  -0.953   0.113   0.043   0.844
E H  2QJBC                 27    0.298  54.133   0.917   0.159   0.516   0.325
B C  2QJBC                 28    0.034   4.731  -0.092   0.087   0.683   0.230
B F  2QJBC                 29    0.075  15.093   0.861   0.085   0.775   0.140
B T  2QJBC                 30    0.126  17.448   0.287   0.085   0.775   0.140
B M  2QJBC                 31    0.103  20.630   0.789   0.085   0.775   0.140
B I  2QJBC                 32    0.073  13.524   0.136   0.011   0.918   0.071
B E  2QJBC                 33    0.224  39.080   0.100   0.011   0.918   0.071
E E  2QJBC                 34    0.268  46.785   0.448   0.011   0.918   0.071
E D  2QJBC                 35    0.283  40.852  -0.010   0.023   0.655   0.322
E D  2QJBC                 36    0.586  84.457  -0.831   0.021   0.279   0.699
E Q  2QJBC                 37    0.764 136.361  -0.902   0.005   0.045   0.951
E G  2QJBC                 38    0.456  35.919  -0.348   0.004   0.085   0.910
E E  2QJBC                 39    0.461  80.484   0.144   0.004   0.616   0.381
E T  2QJBC                 40    0.378  52.373   0.503   0.011   0.918   0.071
E T  2QJBC                 41    0.347  48.115   0.541   0.006   0.962   0.032
B L  2QJBC                 42    0.244  44.750   1.102   0.001   0.959   0.040
E T  2QJBC                 43    0.323  44.800  -0.528   0.001   0.900   0.099
B S  2QJBC                 44    0.220  25.737   0.193   0.003   0.718   0.279
B G  2QJBC                 45    0.227  17.849  -1.669   0.004   0.420   0.576
B C  2QJBC                 46    0.067   9.463  -1.529   0.005   0.336   0.660
B L  2QJBC                 47    0.139  25.396   0.082   0.004   0.197   0.799
E G  2QJBC                 48    0.328  25.806   0.864   0.005   0.045   0.951
E L  2QJBC                 49    0.327  59.800  -0.014   0.018   0.019   0.964
E E  2QJBC                 50    0.655 114.463  -0.936   0.018   0.019   0.964
E G  2QJBC                 51    0.774  60.937  -2.044   0.016   0.005   0.979
E S  2QJBC                 52    0.430  50.455  -1.274   0.018   0.047   0.935
E D  2QJBC                 53    0.498  71.776  -0.316   0.019   0.141   0.840
B F  2QJBC                 54    0.076  15.313  -1.795   0.005   0.336   0.660
B Q  2QJBC                 55    0.264  47.133   0.030   0.023   0.655   0.322
B C  2QJBC                 56    0.026   3.594  -1.336   0.023   0.655   0.322
E R  2QJBC                 57    0.346  79.211   0.046   0.005   0.336   0.660
E D  2QJBC                 58    0.407  58.649  -1.638   0.004   0.138   0.858
E T  2QJBC                 59    0.551  76.465  -0.714   0.018   0.088   0.893
E P  2QJBC                 60    0.434  61.556  -1.394   0.018   0.088   0.893
E I  2QJBC                 61    0.497  91.871  -0.068   0.018   0.047   0.935
E P  2QJBC                 62    0.312  44.301  -1.410   0.018   0.019   0.964
E H  2QJBC                 63    0.505  91.896  -1.063   0.018   0.019   0.964
E Q  2QJBC                 64    0.495  88.353  -0.594   0.018   0.019   0.964
E R  2QJBC                 65    0.550 125.996  -1.335   0.018   0.047   0.935
E R  2QJBC                 66    0.356  81.432   0.080   0.020   0.205   0.775
E S  2QJBC                 67    0.302  35.348   0.395   0.023   0.655   0.322
B I  2QJBC                 68    0.126  23.365   1.090   0.018   0.846   0.136
E E  2QJBC                 69    0.332  58.035   0.963   0.018   0.846   0.136
B C  2QJBC                 70    0.031   4.409   0.113   0.023   0.655   0.322
B C  2QJBC                 71    0.039   5.419   0.511   0.021   0.451   0.528
E R  2QJBC                 72    0.417  95.493   0.097   0.018   0.088   0.893
E T  2QJBC                 73    0.532  73.830  -0.542   0.018   0.019   0.964
E N  2QJBC                 74    0.437  63.933  -0.820   0.058   0.017   0.925
B L  2QJBC                 75    0.225  41.179   0.518   0.115   0.016   0.868
B C  2QJBC                 76    0.053   7.441  -0.613   0.115   0.016   0.868
B N  2QJBC                 77    0.250  36.585  -0.476   0.115   0.016   0.868
E Q  2QJBC                 78    0.526  93.908  -0.065   0.115   0.016   0.868
E Y  2QJBC                 79    0.432  92.383  -0.234   0.053   0.043   0.903
B L  2QJBC                 80    0.105  19.152   0.231   0.018   0.047   0.935
E Q  2QJBC                 81    0.494  88.139   0.736   0.018   0.088   0.893
B P  2QJBC                 82    0.242  34.368  -0.982   0.004   0.085   0.910
E T  2QJBC                 83    0.647  89.711  -0.814   0.005   0.045   0.951
E L  2QJBC                 84    0.286  52.422   0.570   0.005   0.015   0.979
E P  2QJBC                 85    0.771 109.362  -0.624   0.003   0.003   0.994

Explain the output.  Go back.
```

---
